# Influence of Nest Microbiota on Hatching Success of *Caretta Caretta* on Lampedusa Island

**DOI:** 10.1007/s00248-026-02699-1

**Published:** 2026-01-29

**Authors:** Fanny Claire Capri, Elena Prazzi, Giulia Casamento, Rosa Alduina

**Affiliations:** 1 Dipartimento Scienze e Tecnologie Biologiche, Chimiche e Farmaceutiche, University of Palermo, Viale delle Scienze, Palermo, 90133 Italy; 2 National Biodiversity Future Center (NBFC), Piazza Marina, 61, Palermo, 90133 Italy; 3 Legambiente Sicilia- Ente Gestore Riserva Naturale Orientata Isola di Lampedusa, Via Vittorio Emanuele, 25, Lampedusa, AG 92031 Italy; 4 Legambiente Sicilia- Ente Gestore Riserve Naturali, via Paolo Gili,4, Palermo, 90138 PA Italy

**Keywords:** Metabarcoding, *Caretta caretta*, Nest hatching, *Pseudomonas*, *Ochrobactrum*, Chlamydia

## Abstract

Egg hatching success in sea turtle nests can be influenced by multiple abiotic and biotic factors. Although interest in nest microbiota as a determinant of embryo development and viability is increasing, its role has not yet been fully elucidated. In this study, we profiled the bacterial communities of four *Caretta caretta* nests on Lampedusa Island: Cala Pisana (P1 and P2) and Spiaggia dei Conigli (C1 and C2), which showed different hatching success rates (P1 = 85.2%, P2 = 1.1%, C1 = 1.1%, C2 = 0.0%). Using 16S rRNA gene (V3-V4) sequencing, we analyzed different sample types, including sand from inside and outside the nest chamber, eggshells, and inner membranes. Alpha diversity was highest in sand and lower in eggshells and inner membranes. β-diversity clearly separated the only successful nest (P1) from the others (P2, C1, and C2) (PERMANOVA *p* < 0.001). Across all nests, the dominant phyla were Proteobacteria, Firmicutes, Actinobacteriota, and Bacteroidota; notably, the Firmicutes/Bacteroidota (F/B) ratio was lowest in P1 compared with P2, C1, and C2. Nests with low hatching success were Firmicutes-dominated, enriched in *Bacillus* and *Pseudomonas*, and harbored hydrocarbon-degrading genera (*Pseudoxanthomonas* and *Devosia*), suggesting environmental influences. Opportunistic pathogens (*Ochrobactrum* and Simkaniaceae), likely associated with vertical transmission, were detected exclusively in C1 and C2 nests. Overall, our findings highlight the potentially critical role of nest microbiota in reproductive success. Both vertical (maternal) and horizontal (environmental and anthropogenic) transmission appear to shape microbial composition, potentially affecting hatchling viability and offering useful insights for conservation monitoring.

**Supplementary Information:**

The online version contains supplementary material available at 10.1007/s00248-026-02699-1.

## Introduction

Loggerhead sea turtles (*Caretta caretta*) are a bioindicator species of global change, and the Mediterranean population is listed as Least Concern by the IUCN [1].

*Caretta caretta* regularly nests along the Italian coasts, and recent data indicate an increase in nesting events in the Pelagian Islands, Linosa and Lampedusa, with documented nesting activity spanning the last four decades [2, 3]. *C. caretta* follows a reproductive strategy compensating for the high mortality rates encountered throughout its life cycle: females migrate to sandy beaches to lay multiple times per season, typically between two and seven clutches each containing approximately 100 eggs [4]. Numerous biotic and abiotic factors influence the reproductive success of the species. Anthropogenic pressures, such as marine pollution and habitat destruction, along with environmental challenges—including climate change, predation, and extreme weather events— significantly impact both hatching success and hatchling survival rates [5–10].

In recent years, increasing attention has been given to the microbial communities associated with sea turtles due to their potential impact on host health, ecology, and conservation. Research on *C. caretta* microbiota has provided valuable insights into host-microbe interactions, particularly concerning rehabilitation and rescue efforts [11–15]. Despite previous research on turtle-associated microbiota, limited knowledge exists regarding the composition, function, and potential pathogenic role of microbial communities in nests. Understanding these interactions is essential to assessing their impact on hatching success and refining effective conservation strategies.

To date, only a limited number of studies have employed next-generation sequencing (NGS) of the 16S rRNA gene to characterize the microbiota of sea turtle eggs and investigate its correlation with hatching success [16–20]. The egg microbiota can derive from several sources: vertically through the female ovudict and cloaca [21]– [22] and horizontally from the surrounding sand. In addition, environmental factors, such as temperature, humidity, sand grain size, and anthropogenic inputs (*e.g*., chemical pollutants, human organic contamination, and fishing activities) may influence this transmission. These factors collectively shape the microbial community and may ultimately influence hatching success [17, 23]. Despite these findings, the functional roles of microbial communities associated with sea turtle nests and eggs remain largely unknown.

**Fig. 1:**
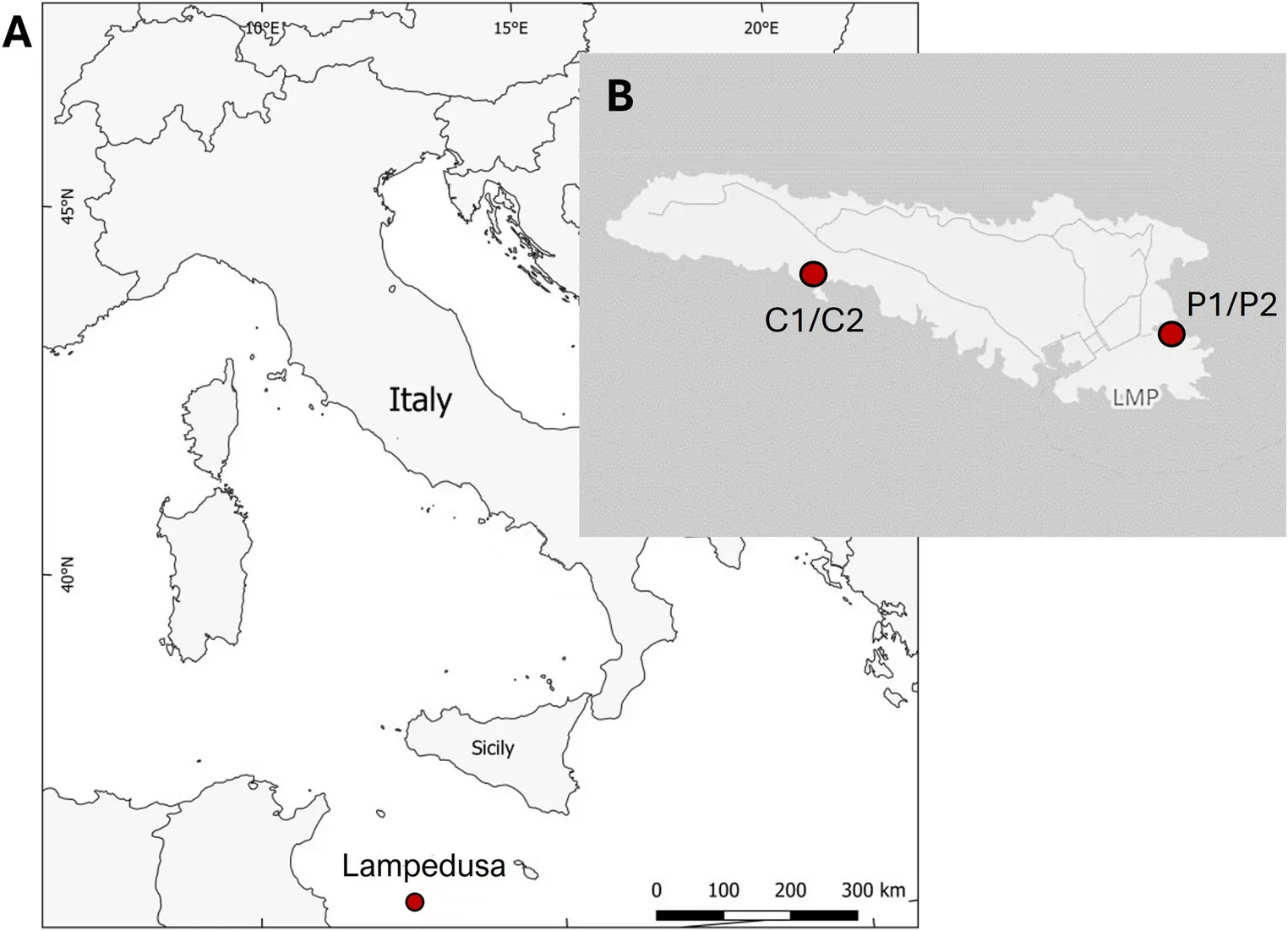
Locations of the samples *C.caretta* nests on Lampedusa Island. **A** Lampedusa Island, indicated by the red circle. **B** The four nests located at Cala Pisana (P1 and P2) and Spiaggia dei Conigli (C1 and C2). Maps were created using QGIS software v.3.6

This study aims to characterize the bacterial community of hatched and unhatched eggs from four *C. caretta* nests located on two beaches in Lampedusa —Cala Pisana (P1 and P2) and Spiaggia dei Conigli (C1 and C2) — which exhibit different hatching success rates (85.2% for P1, 1.1% for P2, 1.1% for C1 and 0.0% for C2. Cala Pisana, the second natural bay of Lampedusa, is located at the far eastern end of the island, approximately 1.5 km from the town center. Spiaggia dei Conigli is situated in the southern part of the island and falls within the Nature Reserve “Isola di Lampedusa” established in 1996 by the Sicilian Region and managed by Legambiente Sicilia. The beach covers an area of 3500 m^2^ and remains isolated from inhabited areas (Fig. 1). By examining the composition and dynamics of microbial communities in nesting environments at these two ecologically distinct beaches, this study seeks to identify potential microbial taxa that may serve as indicators of nest hatching success. The findings will contribute to the development of targeted conservation strategies to mitigate microbial threats and enhance hatching success in *C. caretta* populations.

## Materials and Methods

### Site Description and Sampling

Samples were collected from two nests of the sea turtle *C. caretta* on the beach of Cala Pisana (P1 and P2)(35°30’18” N, 12°37’32” E) and two nests on the Spiaggia dei Conigli (C1 and C2) (35°30’47"N, 12°33’27"E) at Lampedusa Island (Sicily, Italy) during the summer of 2021, under the authorization for the years 2021–2023 of the Ministero della Transizione Ecologica held by Legambiente Sicilia. Figure 1 shows the maps of the nesting beaches.

Approximately 72 h after the last hatchling emerged from nests P1, P2 and C1, the nests were excavate -using sterile disposable gloves- and examined for the collection of five types of material per nest: eggshell fragments from hatched eggs (coded Eg_Hat), unhatched eggs (Eg_Unh), the inner membrane of unhatched eggs (Im), nest sand (Sn_In), and external nest sand (Sn_Out). For nest C2, from which no hatchlings emerged, excavation was performed 76 days after oviposition. Sand samples were collected at a depth of approximately 25–40 cm within the nesting chamber, indicated as “sand inside”. Additionally, the sand surrounding the nest was sampled, referred to as “sand outside”. We collected the actual number of eggs observed per nest, and all samples were placed in separate sterile bags and stored at 4 °C in the field until they were transferred to -20 °C in the laboratory. Before analysis, the egg surface was washed with 15 mL of distilled water to remove loosely attached biofilm. The shells of the unhatched eggs were opened aseptically with a sterile scalpel, and the inner membrane was separated with sterile forceps and transferred to sterile tubes, after which DNA extraction was performed.

For each nest, three biological replicates for each sample type (Eg_Hat, Eg_Unh, Im, Sn_In, and En_Out) were collected and subjected to metagenomic DNA extraction as reported in Sect. 2.2. An equal amount of the three extracted metagenomic DNAs from each sample type was pooled. This approach generated a pooled sample representing the overall microbial community of each sample type per nest. While pooling ensured sufficient DNA yield and sequencing depth, it reduced within-group variability and limited the statistical independence of samples. A total of 18 metagenomic DNA pools were obtained; two pools were not available (*i.e*., Eg_Hat in nest C2, because no eggs were hatched, and Im in nest P2, due to advanced egg decomposition that prevented processing of the inner membrane). The details of the samples analyzed are summarized in Table 1.

**Table 1:** Description of the samples analyzed in this study

Nest	Location	Number of hatched eggs	Hatching success (%)	Sample	Brief Description
**P1**	Cala Pisana	69/81	85.2%	Sn_Out_P1	Sand outside the nest
				Sn_In_P1	Sand inside the nest
				Eg_Hat_P1	Shells of hatched eggs
				Eg_Unh_P1	Shells of unhatched eggs
				Im_P1	Inner membranes of unhatched eggs
**P2**	Cala Pisana	1/110	1.1%	Sn_Out_P2	Sand outside the nest
				Sn_In_P2	Sand inside the nest
				Eg_Hat_P2	Shells of hatched eggs
				Eg_Unh_P2	Shells of unhatched eggs
**C1**	Spiaggia dei Conigli	1/91	1.09%	Sn_Out_C1	Sand outside the nest
				Sn_In_C1	Sand inside the nest
				Eg_Hat_C1	Shells of hatched eggs
				Eg_Unh_C1	Shells of unhatched eggs
				Im_C1	Inner membranes of unhatched eggs
**C2**	Spiaggia dei Conigli	0/63	0%	Sn_Out_C2	Sand outside the nest
				Sn_In_C2	Sand inside the nest
				Eg_Unh_C2	Shells of unhatched eggs
				Im_C2	Inner membranes of unhatched eggs

### Genomic DNA extraction, PCR amplification, and Sequencing

The eighteen samples listed in Table 1 were subjected to metagenomic DNA extraction using the protocol reported in [19]. Metagenomic DNA was verified by electrophoresis on 1% w/v agarose gel. The purity and quantity of DNA were assessed using a NanoDrop 2000c spectrophotometer (Thermo Fisher Scientific, MA, USA). An equal amount of extracted metagenomic DNA from each sample type (*n* = 3) was pooled. A 464 bp fragment corresponding to the V3-V4 region of the 16 S rRNA gene was amplified using the primers Pro341-F (CCTACGGGNBGCASCAG) and Pro805R (GACTACNVGGGTATCTAATCC) [24]. Amplification products were sequenced in one 300-bp paired-end run on an Illumina MiSeq platform at BMR Genomics s.r.l. (Padova, Italy).

### Raw Data Processing and Statistical Analyses

**Fig. 2:**
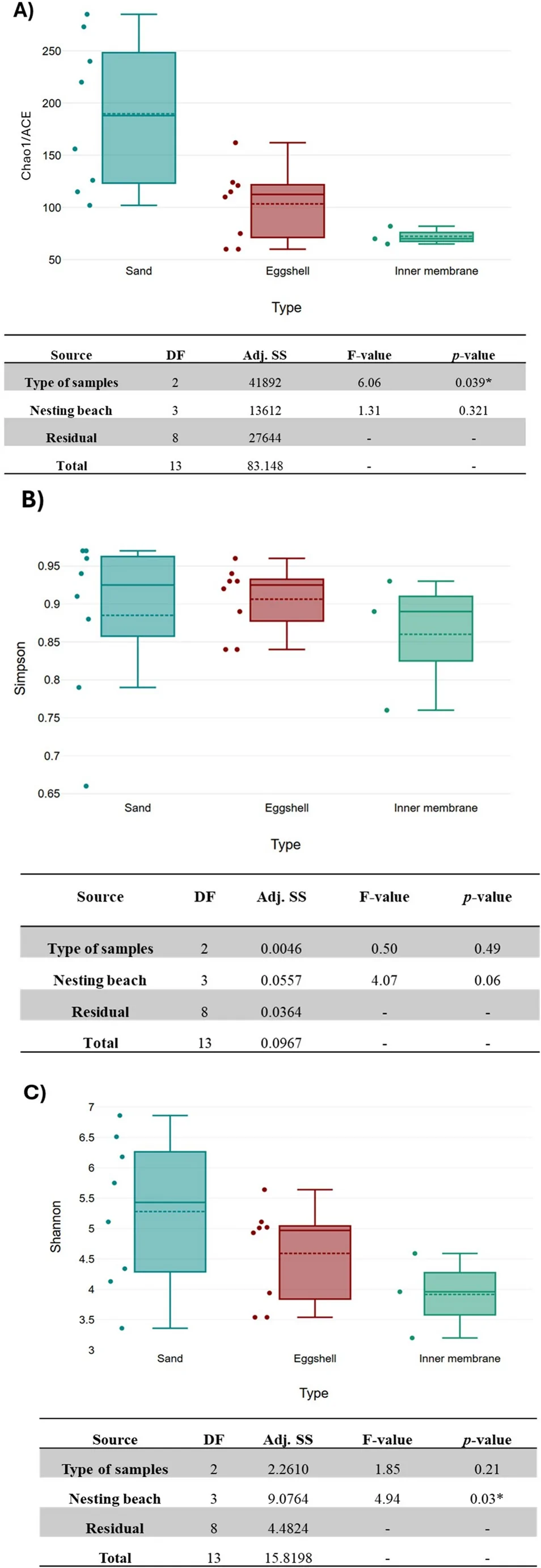
Microbial α-diversity across sample types: Chao1/ACE (**A**), Simpson (**B**), and Shannon (**C**) Indices with ANOVA results. DF: Total degrees of freedom; Adj. SS: Adjusted sums of squares; *p*-value with * indicates significance (p*-value* ≤ 0.05)
  The Shannon index revealed greater differences between the nesting beaches (*p-value* = 0.03) than eggshells and inner membrane (Fig. 2C). Specifically, Sn_out_C1 had a Shannon index of 4.34, compared to Sn_out_P1 with a Shannon index of 6.51 (Fig. 2C). Hatched eggshells (Eg_Hat) showed higher average diversity values than unhatched eggshells (Eg_Unh), as observed in P1 (Eg_Hat_P1, Shannon = 5.64 vs. Eg_Unh_P1, Shannon = 5.01) and C1 (Eg_Hat_C1, Shannon = 5.02 vs. Eg_Unh_C1, Shannon = 3.94).
- β-diversity
  Based on β-diversity metrics, the PCoA analysis of the four *C. caretta* nests showed a clear separation between the microbial profiles of nests P1, P2, C1, and C2 (Fig. 3). The samples from nest P1, the only nest with a high hatching success, exhibited a significantly different microbiota composition compared to those from nest P2 (orange). Additionally, nests C1 and C2 (with low hatching success, indicated by the blue color) cluster more closely together, indicating an overlap in microbial community composition. Multivariate PERMANOVA statistical analyses revealed significant differences in microbial communities among the four nests (permutation test, *p* ≤ 0.001, 999 permutations).

The raw 16S rRNA data were processed using the QIIME2 suite [25] as paired-end sequences. The de-noising approach processed overlapping paired-end reads using the DADA2 plug-in (Table S1) [26]. Unique Amplicon Sequence Variants (ASVs) were assigned and aligned to the Silva 138.2 reference database at 99% sequence similarity. QIIME2 generated rarefaction curves, Good’s coverage index, and alpha diversity metrics (Fig. 2 and Table S2). Rarefaction analysis was conducted by plotting the number of observed ASVs against the filtered reads for each sample. The number of ASVs and the relative abundances of different taxonomic levels were determined. Based on the rarefaction curve, the alpha diversity metrics were calculated on a rarefied frequency-feature table. Principal Coordinates Analysis (PCoA) was performed in the software Emperor, starting from the previously transformed Jaccard distance matrix using the square root transformation. PERMANOVA (Permutational Multivariate Analysis of Variance) was performed on the Jaccard distance matrix to assess differences in overall microbial community structure among nests. Each pooled DNA sample (*n* = 18 pools) was treated as one independent sample. The model included only “Nest” as the grouping factor, without interaction terms, to avoid overparameterization. Significance was assessed using 999 permutations. PERMANOVA results should be interpreted as indicating broad community-level patterns rather than as strong inferential tests.

Expression Heat Maps were conducted using the online web server (http://heatmapper.ca/expression/) based on bacterial genus signatures. Two-way ANOVA (Analysis of Variance) was performed to test for differences in alpha-diversity among sample types and nesting beaches. Each pooled DNA sample (*n* = 18 pools) was treated as one independent sample. The model included two fixed and orthogonal factors: “Type” (three levels: sand, eggshell, inner membrane), “Nesting beach” (four nests, two from Cala Pisana and two nests of Spiaggia dei Conigli). Because statistical analyses were conducted on pooled samples with limited replication, multiple-testing correction was not applied. Therefore, ANOVA results are used as descriptive indicators rather than inferential tests.

The sequence dataset was deposited in the GenBank database (BioProject ID: PRJNA1289694).

## Results

### Analysis of microbial diversity and richness

- α-diversity
  A total of 594,538 high-quality reads were obtained from the 1,321,071 raw reads. After filtration, denoising, and merging, 1,365 ASVs were identified using the QIIME2 suite. The rarefaction curves, comparing ASV abundances and sequence counts, showed that the analyses accurately reflected the communities under investigation, as confirmed by Good’s coverage index (an average of 1 across all samples) (Table S2).
  Observed ASVs and α-diversity indices (Chao1 and ACE, Fig. 2A) revealed higher microbial richness in the nest sand compared to the eggshell and inner membrane. Specifically, the sand outside the nest showed higher microbial richness (Chao1 and ACE) than the sand inside the nest (e.g., Sn_Out_P1 = 285 to Sn_in_P1 = 102; Sn_Out_P2 = 273 to Sn_in_P2 = 220; Sn_Out_C1 = 156 to Sn_In_C1 = 115; Sn_Out_C2 = 240 to Sn_In_C2 = 126, Table S2). Simpson’s index, although not statistically significant, confirmed a higher microbial diversity in the sand samples (Fig. 2B).

**Fig. 3:**
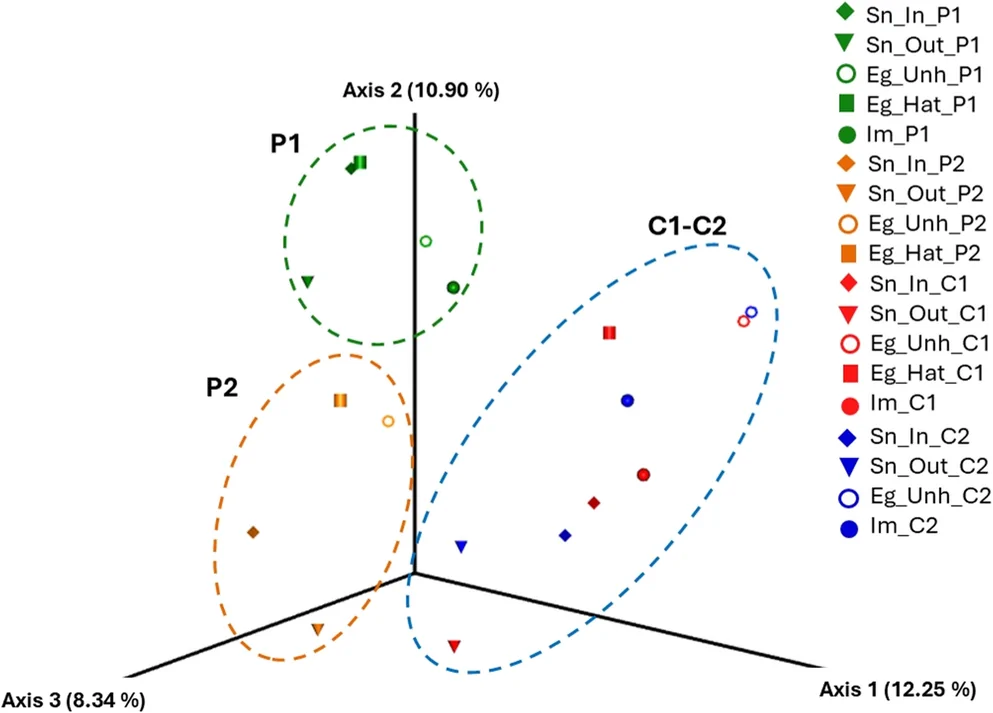
The Principal Coordinates Analysis (PCoA) plot was measured using the Jaccard distance between two nests of Cala Pisana (P1 in green and P2 in orange) and two nests of Spiaggia dei Conigli (C1 and C2 in blue)

### Microbial Composition and Biodiversity by 16S rRNA Gene Metabarcoding

As an overview, the taxonomic composition of the microbial communities associated with *C. caretta* nests at Cala Pisana (P1, P2) and Spiaggia dei Conigli (C1, C2) revealed distinct patterns of bacterial dominance across all sample types within each nest (Fig. S1). The most abundant phyla across all four nests analyzed were Proteobacteria, Firmicutes, Actinobacteriota, and Bacteroidota. Bacteria belonging to other phyla, such as Verrucomicrobiota and Dependentiae, were minor components, if present. The microbial community in P1 was dominated by Proteobacteria (38%) and Bacteroidota (25%) in all the sample types analyzed. In contrast, P2, C1, and C2 showed a more diverse distribution among samples. In comparison, a high abundance of Actinobacteriota (25%−17%) was observed only in the nests from Spiaggia dei Conigli (C1 and C2). To better characterize microbial distribution, we analyzed taxonomic bacterial profiles separately for each sample type (sand, eggshell, and inner membrane).

**Fig. 4:**
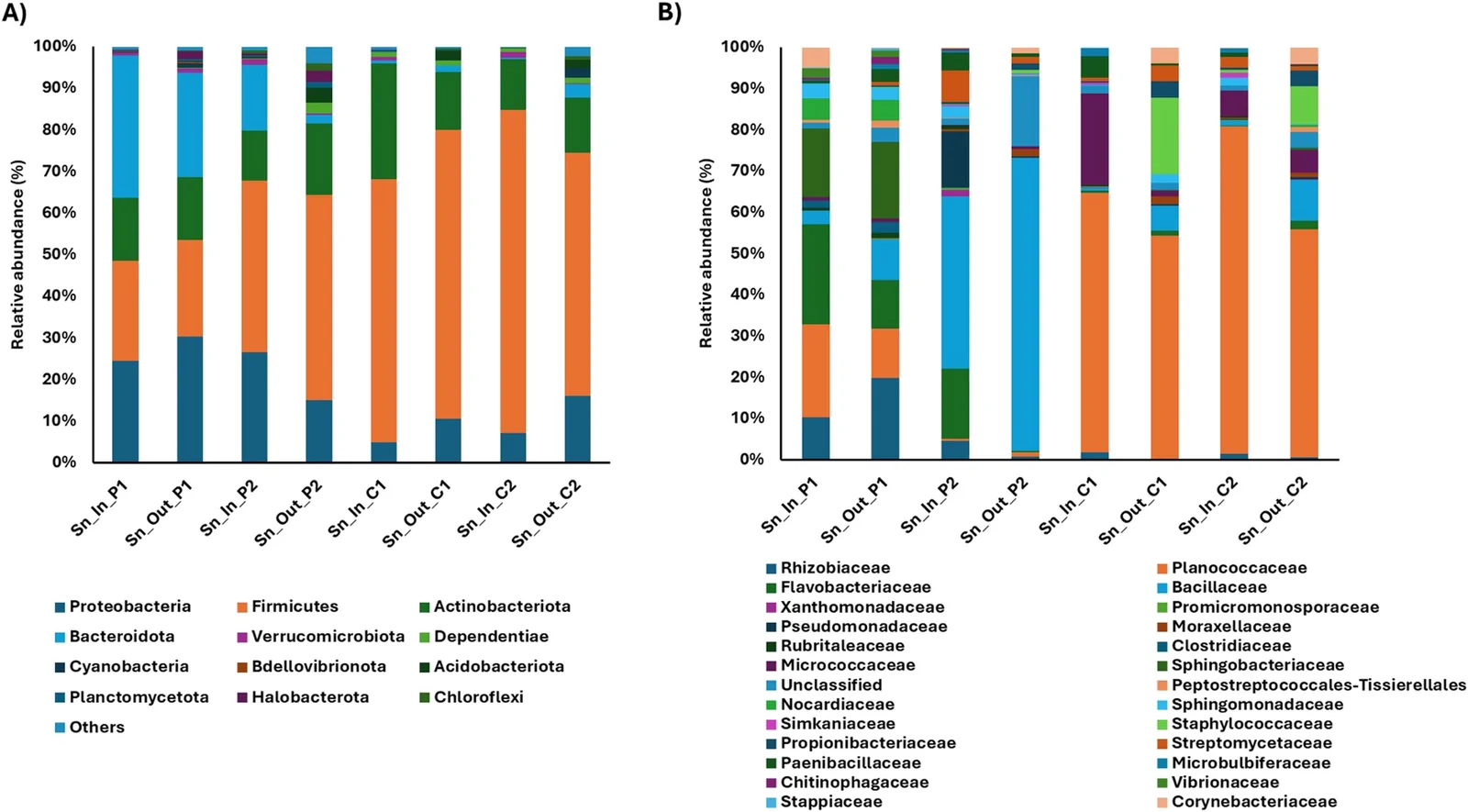
Relative abundance (%) of phyla (**A**) and the main 25 bacterial families (**B**) found in sand inside and outside the four nests collected from Cala Pisana (P1 and P2) and Spiaggia dei Conigli (C1 and C2)
  At the family level (Fig. 4B), P1 was characterized by a higher relative abundance of Rhizobiaceae (8–15%), Flavobacteriaceae (9–20%), and Sphingobacteriaceae (14%) in contrast to low-hatching nests (P2, C1, and C2). P2 was characterized by a high presence of Bacillaceae (37–47%), while C1 and C2 were dominated by the family Planococcaceae (40–70%), together with an increase of Micrococcaceae in the sand inside nests C1 (20%) and C2 (6%) and of Staphylococcaceae in the sand outside nests C1 (16%) and C2 (7%).
- Microbial composition of eggshell samples
  Proteobacteria were abundant in eggshells, and in only two samples (i.e., Eg_Unh_P1 = 41% and Eg_Hat_C1 = 37%) there was an abundance of Firmicutes (Fig. 5A). The main difference between nests was characterized by a significant abundance of Actinobacteriota in unhatched eggs in nests in P2, C1, and C2 (Eg_Unh_P2 = 18%; Eg_Unh_C1 = 27%; Eg_Unh_C2 = 36%) compared to hatched eggs and eggshells from P1. While P1 was characterized by an abundance of Bacteroidota in hatched eggs (Eg_Hat_P1 = 38%), the other nests showed a strong decrease in this phylum (Eg_Hat_P2 = 11%, Eg_Hat_C1 = 9%). Each nest was characterized by a different percentage of bacterial strains: Rhizobiaceae and Flavobacteriaceae were more abundant in eggshells from P1 of Cala Pisana, while Pseudomonadaceae were more abundant in nest P2. Bacillaceae and Planococcaceae were mainly present in nest C1, while Promicromonosporaceae were present in both C1 and C2 of Spiaggia dei Conigli (Fig. 5B).

- Microbial composition of sand samples
  In P1 nest sand, no single phylum predominated: Proteobacteria (25–30%), Firmicutes (23–34%), and Bacteroidota (25–34%) were of comparable abundance (Fig. 4A). By contrast, sand from P2, C1, and C2 were dominated by Firmicutes (41–77%) and depleted in Bacteroidota (1–15%) and Proteobacteria (5–26%).

**Fig. 5:**
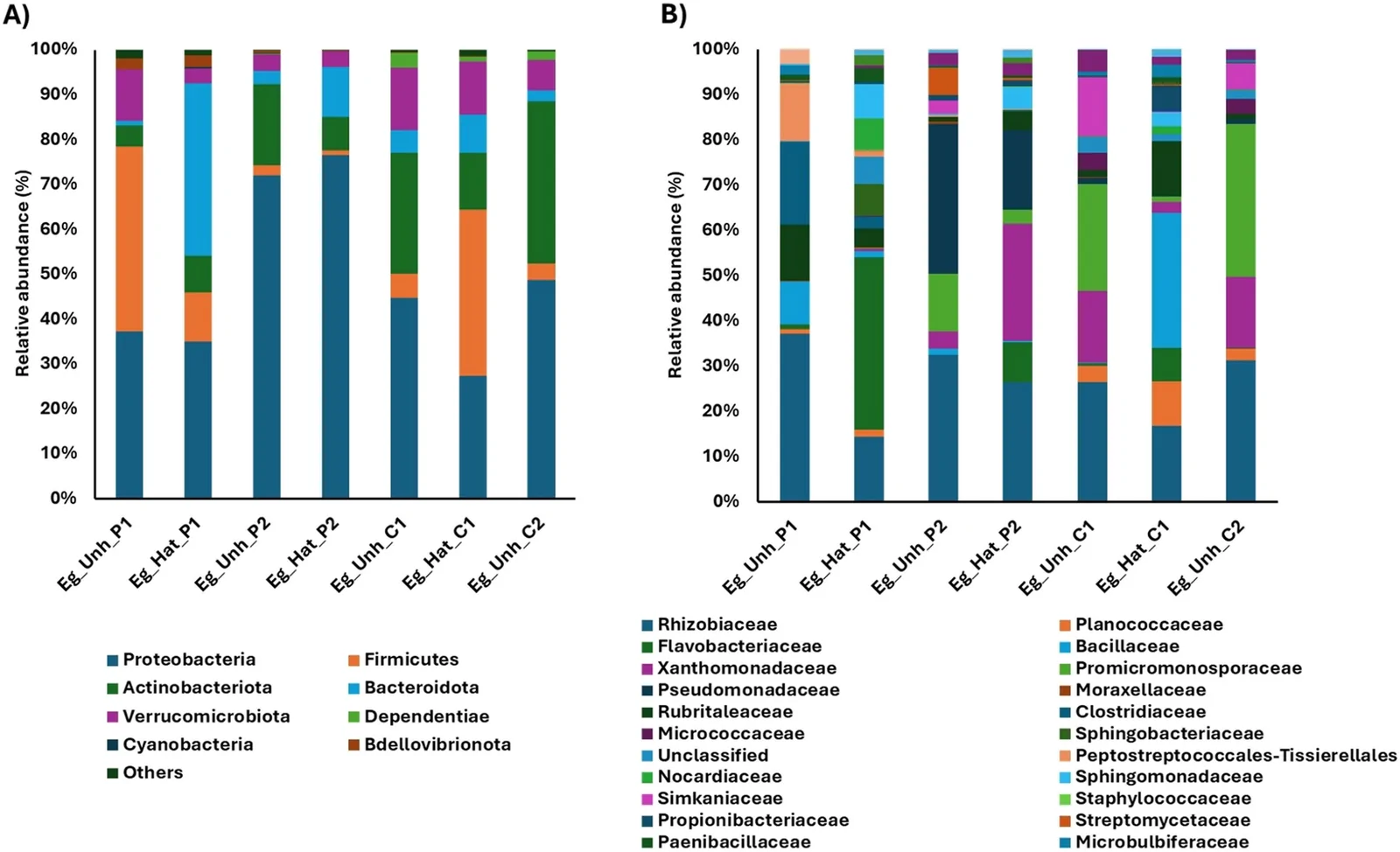
Relative abundance (%) of phyla (**A**) and the main 25 bacterial families (**B**) from unhatched and hatched eggshells collected from the four nests at Cala Pisana (P1 and P2) and Spiaggia dei Conigli (C1 and C2)
- Microbial composition of inner membrane samples
  The inner membrane of the eggs was characterized by a high presence of Proteobacteria in all nests (Fig. 6 A). At the same time, an abundance of Firmicutes was detected only in the inner membrane of the eggs of nest P1 (Im_P1 = 26%). Within the inner membrane of the eggs from beaches C1 and C2, with a hatching success of nearly 0%, there was a decrease in Firmicutes (Im_C1 = 4%; Im_C2 = 3%). In particular, only in the sample Im_C1 there was an increase in Actinobacteriota (Im_C1 = 44%), as well as in Xanthomonadaceae (Im_C1 = 37%) and Promicromonosporaceae (Im_C1 = 36%) at the family level (Fig. 7B). The family Pseudomonadaceae was abundant only in the inner membrane of the unhatched eggs of the nest C2 (Im_C2 = 16%).

**Fig. 6:**
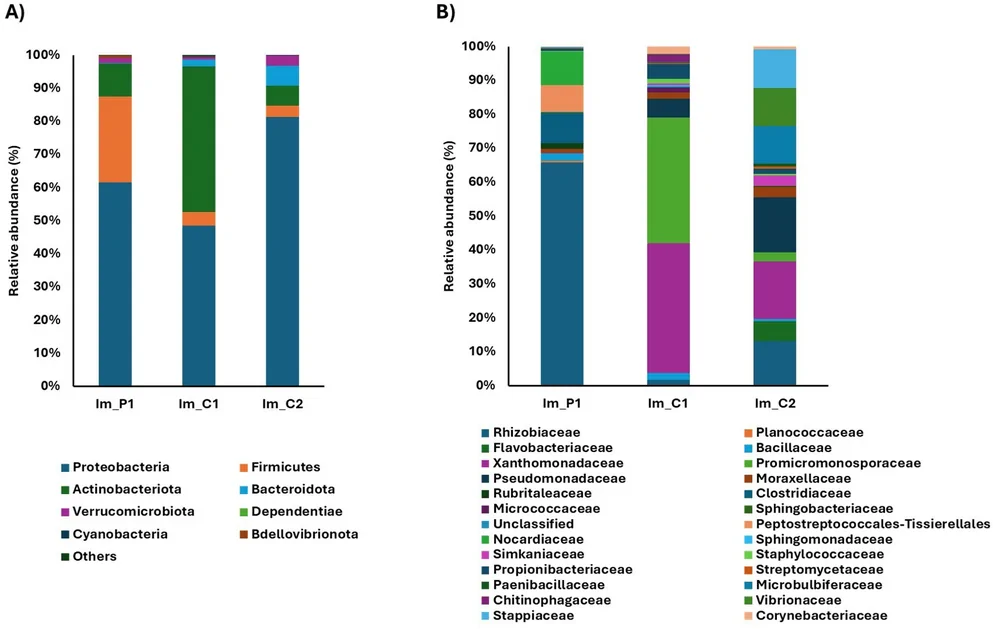
Relative abundance (%) of phyla (**A**) and the main 25 bacterial families (**B**) found in the inner membranes of unhatched eggs from the four nests collected from Cala Pisana (P1 and P2) and Spiaggia dei Conigli (C1 and C2)

### Specific Genera Signatures in *C. caretta* Nests

**Fig. 7:**
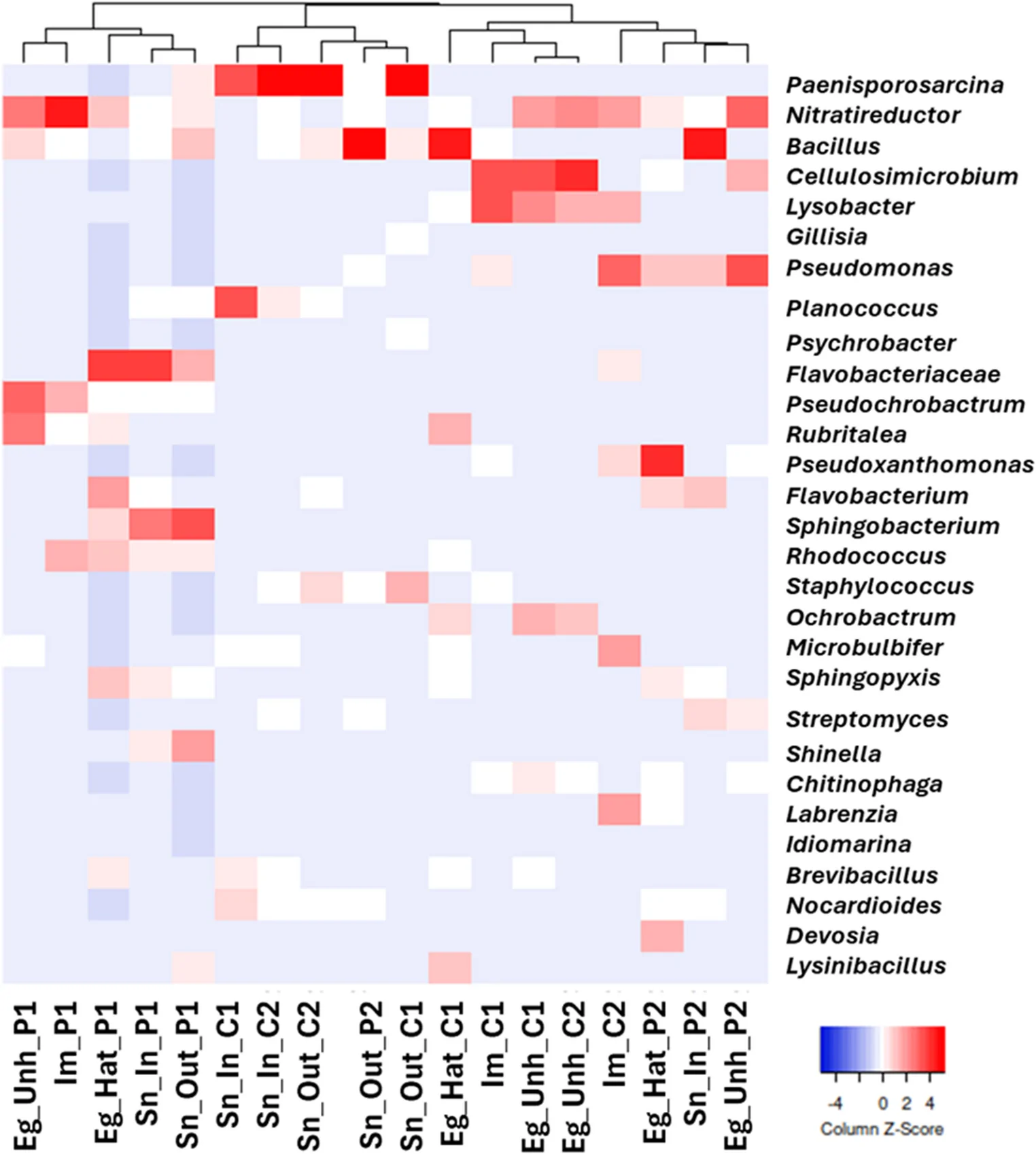
Heatmap of the most abundant bacterial genera generated by “complete linkage’’ calculation using Spearman’s rank correlation. The color scale, from blue to red, represents the relative values of the abundance of each genus

Heatmap analysis (Fig. 7) confirmed that the bacterial genera of the nest P1 created a distinct cluster, while P2, C1, and C2 were subclustered, sharing a similar genus composition.

In nest P2, an abundance of *Bacillus* was recorded in the sand inside the nest (Sn_In_P2) and *Pseudomonas*, *Pseudoxanthomonas*, and *Devosia* in the hatched and unhatched eggshells. Different bacterial signatures were detected in nests from Spiaggia dei Conigli. An abundance of *Paenisporosarcina* was detected in the Sn_In_C1 and C2, as well as in the Sn_Out_C1 and C2. In contrast, the unhatched eggshells and their inner membranes were characterized by an abundance of *Cellulosimicrobium*, *Lysobacter*, and *Ochrobactrum*.

## Discussion

This study investigated the microbial communities of four *C. caretta* nests laid at Cala Pisana (P1 and P2) and Spiaggia dei Conigli (C1 and C2), on Lampedusa Island (Sicily, Italy). To date, only three studies have analyzed the nest microbiota of *C. caretta* using metagenomic DNA approaches [18–20]. Findings of these studies suggested that both maternal and environmental factors, together with the protective role of the eggshell, shape the egg microbiota of sea turtles [18–20]. We analyzed the bacterial composition of sand, eggshells, and inner membrane from four nests featuring different hatching success rates (P1 = 85%, P2 = 1%, C1 = 1%, and C2 = 0%), located on two beaches with different environmental characteristics, in order to assess potential bacterial influences on embryo development.

Our results clearly showed higher microbial richness in sand samples than in eggshells and inner membranes (Fig. 2). Microbial diversity was generally higher in the sand outside the nests than within, supporting previous findings that incubation conditions selectively shape microbial communities [16, 19, 20, 27]. Hatched eggshells consistently exhibited greater microbial diversity than unhatched ones, reinforcing the hypothesis that a balanced and functionally diverse microbiota may benefit embryonic development. A clear decrease in microbial richness and diversity was observed from sand to eggshells to inner membranes, supporting the concept of the eggshell acting as a biological “filter” that, when functioning properly, prevents colonization by pathogenic species capable of compromising embryo development [17, 21, 28].

Our findings revealed significant differences in microbial composition between the single high-hatching nest (P1) and the three low-hatching nests (P2, C1, and C2), as demonstrated by β-diversity analyses (Fig. 3). Notably, although nests P1 and P2 were located on the same beach, their distinct microbiota profiles suggest that localized microenvironmental or anthropogenic factors may act on the microbial colonization of the nest, as also stated by previous reports [17, 29, 30]. However, a maternal effect cannot be ruled out, as the oviposition dates suggest different mothers. The predominant bacterial phyla across all four nests were Proteobacteria, Firmicutes, Actinobacteriota, and Bacteroidota, consistent with previous findings from turtle nests in the Mediterranean [18, 19] and other regions worldwide [20, 27]. Notably, higher abundances of Firmicutes were detected in the sands of the nests from Spiaggia dei Conigli. The Firmicutes/Bacteroidota (F/B) ratio is considered a relevant marker of gut dysbiosis, with healthy populations showing a median value of 1 and no higher than 5 [31, 32]. A clear difference in F/B ratios was observed among the four nests, with higher ratios in C1 (mean F/B = 9) and C2 (mean F/B = 12), followed by P2 (mean F/B = 2.8) and P1 (mean F/B = 1.25); however, because this metric originates from mammalian gut studies, its ecological meaning in sand or eggs remains uncertain. Unlike the sand outside the nest, the sand inside P1 was enriched with Bacteroidota, suggesting potential maternal transmission through the oviduct or cloaca [33]. Given their roles in organic matter degradation and symbiotic interactions, the reduced abundance of Bacteroidota in failed nests may indicate a loss of beneficial microbial contributions to egg incubation [20].

- Cala Pisana nests
  Cala Pisana lies outside both the terrestrial reserve and the marine protected area, is located near the inhabited center of Lampedusa, adjacent to access roads and residential buildings, and is therefore subject to substantially higher anthropogenic pressure.
  Despite being deposited on the same beach, the two nests at Cala Pisana exhibited markedly different hatching success rates. These differences could reflect maternal origin, as different females may have laid the two nests. Both hatched and unhatched eggs from nest P1 had a microbiota distinct from that of samples from P2, suggesting that P1 eggs harbor an inner microbial community distinct from their surrounding environment. Indeed, the dominant taxa in P1 eggs belonged to the families Sphingobacteriaceae and Rubritaleaceae, previously reported as typical members of oviparous vertebrate egg microbiota [34] and the carapace microbiota of nesting female sea turtles [35], supporting vertical (maternal) transmission. In contrast, sand inside and outside the P2 nest showed a high abundance of Firmicutes, specifically Bacillaceae, which may indicate environmental alterations at the nesting site or anthropogenic influences [19]; further investigation is needed to determine this.
  At the genus level, *Bacillus* sp., *Pseudomonas* sp., *Pseudoxanthomonas* sp., and *Devosia* sp. were dominant in both sand inside and outside the nest as well as in hatched and unhatched eggshells. *Pseudomonas* and *Bacillus* have previously been linked to hatching failure in sea turtle eggs [19, 20, 23, 36–38]. The presence of *Pseudoxanthomonas* and *Devosia*, known for hydrocarbon and xenobiotic degradation [39]– [40], may suggest environmental contamination. Although the timing of contamination remains uncertain, the ability of these genera to produce proteolytic enzymes [41]– [42], such as proteases, may have compromised eggshell integrity, facilitating pathogen infiltration. Follow-up observations supported these findings: in 2024, a nest laid in the same area as P1 exhibited 83.9% hatching success, whereas a nest deposited near P2 showed only 2% hatching success, confirming the site-specific pattern previously observed.
- Spiaggia dei Conigli nests
  Spiaggia dei Conigli is located within a strictly protected Natural Reserve and Marine Protected Area, characterized by rigorous conservation measures. The surrounding marine area is designated as a no-entry zone for boats and other activities, except regulated daytime bathing, and tourist access to the beach is strictly limited and continuously monitored [43]. Under these conditions, significant anthropogenic contamination is considered unlikely; however, we identified several bacterial signatures that may influence nest hatching failure.
  The similarity of the bacterial communities in C1 and C2, including the abundance of Planococcaceae, Staphylococcaceae, and Actinobacteriota as well as the presence of *Ochrobactrum* and Simkaniaceae, may suggest that probably both nests were deposited by the same female, underscoring the role of maternal imprinting on nest microbiota. Although no female has been sighted at Spiaggia dei Conigli, the deposition dates suggest it may be the same female. This hypothesis is based on the assumption that C1 was laid on August 1 st, 2021, and C2 on August 18th, 2021, an interval of 17 days, which falls within the typical internesting interval (13–17 days) for loggerhead sea turtles [44]. Both C1 and C2 nests, located at Spiaggia dei Conigli, exhibited a significant abundance of Planococcaceae and Staphylococcaceae, as previously reported in eggshells from the Linosa nest [18], along with Actinobacteriota, particularly in unhatched eggshells and inner membranes. Members of Actinobacteriota are key components of soil microbiota and are known producers of antimicrobial compounds [45]. This phylum may represent a microbial signature of Spiaggia dei Conigli; however, its overrepresentation in unhatched eggs raises concerns about a potential impact on embryo viability. Specifically, *Cellulosimicrobium sp.*, detected as a microbial signature in unhatched eggshells and inner membranes of C1 and C2 (Fig. 7), produces hydrolytic enzymes capable of degrading proteins and lipids [46], which may alter eggshell permeability and compromise embryonic development.
  Additionally, the detection of *Dependentiae*, a phylum of obligate endosymbionts with parasitic tendencies [47], may have further influenced embryo viability by host-microbe interactions, altering biofilm formation, or triggering immune responses [48]. The high abundance of *Ochrobactrum* and Simkaniaceae, detected exclusively in C1 and C2 nests, suggests a potential association with nest environments. *Ochrobactrum* is an opportunistic pathogen increasingly associated with severe infections [49]; this represents only the second recorded detection of this genus in sea turtle eggs, which may suggest its role in nest health monitoring and conservation management [19]. Simkaniaceae, a bacterial family within the Chlamydia phylum, includes pathogens and parasites that infect mammals. Although *C. caretta* individuals positive for *Chlamydiae* showed no clinical signs in a previous study [50], these bacteria have been reported to cause lesions and symptoms that can lead to mortality in some marine turtle species [51]– [52].
  It is also noteworthy that sea turtles may act as reservoirs and disseminators of zoonotic agents, such as *Chlamydiae*, potentially posing a risk to both humans and sea turtle populations, including adult and their eggs.
  Subsequent observations at Spiaggia dei Conigli further confirmed the high reproductive suitability of this nesting site. In 2023, a natural (non-relocated) nest laid in the same area as C1 and C2 achieved a hatching success of 96.7%. Moreover, in 2025, six relocated nests laid in the same area exhibited hatching success rates exceeding 80% (range: 81.4–98.6%).
  Some limitations of this study should be acknowledged. Differences observed between nesting beaches were discussed in relation to environmental or anthropogenic factors; however, detailed environmental parameters such as sand grain size, moisture content, temperature profiles, organic matter, and indicators of human disturbance were not measured during sampling. The absence of these data limits our ability to attribute differences in microbial communities between beaches to specific ecological or anthropogenic factors. Future studies integrating environmental measurements, could be useful to clarify the ecological roles and contributions of nest-associated microbiota.

## Conclusions

The analysis of bacterial communities in *C. caretta* nests may represent a valuable tool for sea turtle conservation and for improving hatching success. This study focused on four *C. caretta* nests from Cala Pisana and Spiaggia dei Conigli (Lampedusa, Italy), which exhibited markedly different hatching success rates (P1 = 85%, P2 = 1%, C1 = 1%, C2 = 0%). Our results indicated that the successful nest (P1) was characterized by a low Firmicutes/Bacteroidota ratio and by the presence of bacterial families typically associated with oviparous vertebrates. In contrast, the low-hatching nest (P2) was enriched in Firmicutes, particularly the genera *Bacillus* and *Pseudomonas*, likely derived from the sand outside the nest. In nests C1 and C2, we detected *Ochrobactrum* and *Simkaniaceae*, which may have been major contributors to hatching failure, possibly linked to vertical transmission from the mother. Overall, our findings highlight the crucial role of the nest microenvironment in shaping a specific microbial signature, influenced by both environmental conditions and maternal microbiota.

Future research should integrate metagenomic and transcriptomic approaches to elucidate the functional potential of these microbial communities. Furthermore, evaluating the impacts of climate change and anthropogenic disturbances on nest microbiota could provide critical insights for developing conservation strategies aimed to improve the reproductive success of *C. caretta*.

## Supplementary Information

Below is the link to the electronic supplementary material.

Supplementary Material 1 (DOCX 164 KB)

## Data Availability

The dataset analyzed in the current study are available in the Genbank database, with the following accession numbers BioProjectID: PRJNA1289694.
