# Supplementary material for: Influence of Nest Microbiota on Hatching Success of Caretta Caretta on Lampedusa Island
Source: Microb Ecol. 2026 Jan 29;89(1):51. doi: 10.1007/s00248-026-02699-1 (PMC12904927; doi:10.1007/s00248-026-02699-1)
Supplement: Supplementary file 1 — Supplementary Material 1 (DOCX 164 KB) [file 248_2026_2699_MOESM1_ESM.docx]

**Supplementary Material**

**Table S1.** DADA2 analysis of the samples used in this study.

| Type | Samples | Input | Filtered | Denoised | Merged | Non-chimeric |
| --- | --- | --- | --- | --- | --- | --- |
| Sand | Sn_In_P1 | 29924 | 29250 | 26533 | 25832 | 22890 |
|  | Sn_Out_P1 | 100483 | 50210 | 47615 | 42850 | 41902 |
|  | Sn_In_P2 | 63669 | 32276 | 31172 | 27880 | 26941 |
|  | Sn_Out_P2 | 56805 | 29986 | 28444 | 23904 | 22866 |
|  | Sn_In_C1 | 48252 | 29513 | 28641 | 25838 | 23506 |
|  | Sn_Out_C1 | 41280 | 25389 | 25191 | 24472 | 24446 |
|  | Sn_In_C2 | 65012 | 34409 | 33725 | 27934 | 23822 |
|  | Sn_Out_C2 | 113788 | 31481 | 31172 | 27678 | 26620 |
| Eggshell | Eg_Hat_P1 | 111154 | 47343 | 46041 | 43552 | 42920 |
|  | Eg_Hat_P2 | 55298 | 37628 | 37169 | 35789 | 35520 |
|  | Eg_Hat_C1 | 58971 | 36118 | 33711 | 27934 | 23822 |
|  | Eg_Hat_C2 | 63625 | 27565 | 26950 | 24857 | 20667 |
|  | Eg_Unh_P1 | 86860 | 49322 | 48601 | 46752 | 45736 |
|  | Eg_Unh_P2 | 70347 | 34838 | 34418 | 32801 | 32480 |
|  | Eg_Unh_C1 | 62973 | 37265 | 36474 | 34145 | 31568 |
| Inner membrane | Im_P1 | 62659 | 35568 | 35283 | 34728 | 34421 |
|  | Im_C1 | 46468 | 26100 | 25990 | 25652 | 25490 |
|  | Im_C2 | 63517 | 30630 | 30244 | 29084 | 28712 |

**Table S2.** Descriptions and diversity indices of the samples used in this study.

| Type | Samples | ASV | Chao1 | Simpson | Shannon | ACE | Good’s coverage |
| --- | --- | --- | --- | --- | --- | --- | --- |
| Sand | Sn_In_P1 | 102 | 102 | 0.94 | 5.11 | 102 | 1 |
|  | Sn_Out_P1 | 285 | 285 | 0.97 | 6.51 | 285 | 1 |
|  | Sn_In_P2 | 220 | 220 | 0.96 | 6.18 | 220 | 1 |
|  | Sn_Out_P2 | 273 | 273 | 0.97 | 6.86 | 273 | 1 |
|  | Sn_In_C1 | 115 | 115 | 0.88 | 4.13 | 115 | 1 |
|  | Sn_Out_C1 | 156 | 156 | 0.79 | 4.34 | 156 | 1 |
|  | Sn_In_C2 | 126 | 126 | 0.66 | 3.36 | 126 | 1 |
|  | Sn_Out_C2 | 240 | 240 | 0.91 | 5.75 | 240 | 1 |
| Eggshell | Eg_Hat_P1 | 162 | 162 | 0.96 | 5.64 | 162 | 1 |
|  | Eg_Hat_P2 | 124 | 124 | 0.93 | 5.11 | 124 | 1 |
|  | Eg_Hat_C1 | 115 | 115 | 0.92 | 5.02 | 115 | 1 |
|  | Eg_Hat_C2 | 60 | 60 | 0.84 | 3.54 | 60 | 1 |
|  | Eg_Unh_P1 | 110 | 110 | 0.94 | 5.01 | 110 | 1 |
|  | Eg_Unh_P2 | 121 | 121 | 0.93 | 4.93 | 121 | 1 |
|  | Eg_Unh_C1 | 75 | 75 | 0.89 | 3.94 | 75 | 0.99 |
| Inner membrane | Im_P1 | 65 | 65 | 0.89 | 3.96 | 65 | 1 |
|  | Im_C1 | 70 | 70 | 0.76 | 3.20 | 70 | 1 |
|  | Im_C2 | 82 | 82 | 0.93 | 4.59 | 82 | 1 |


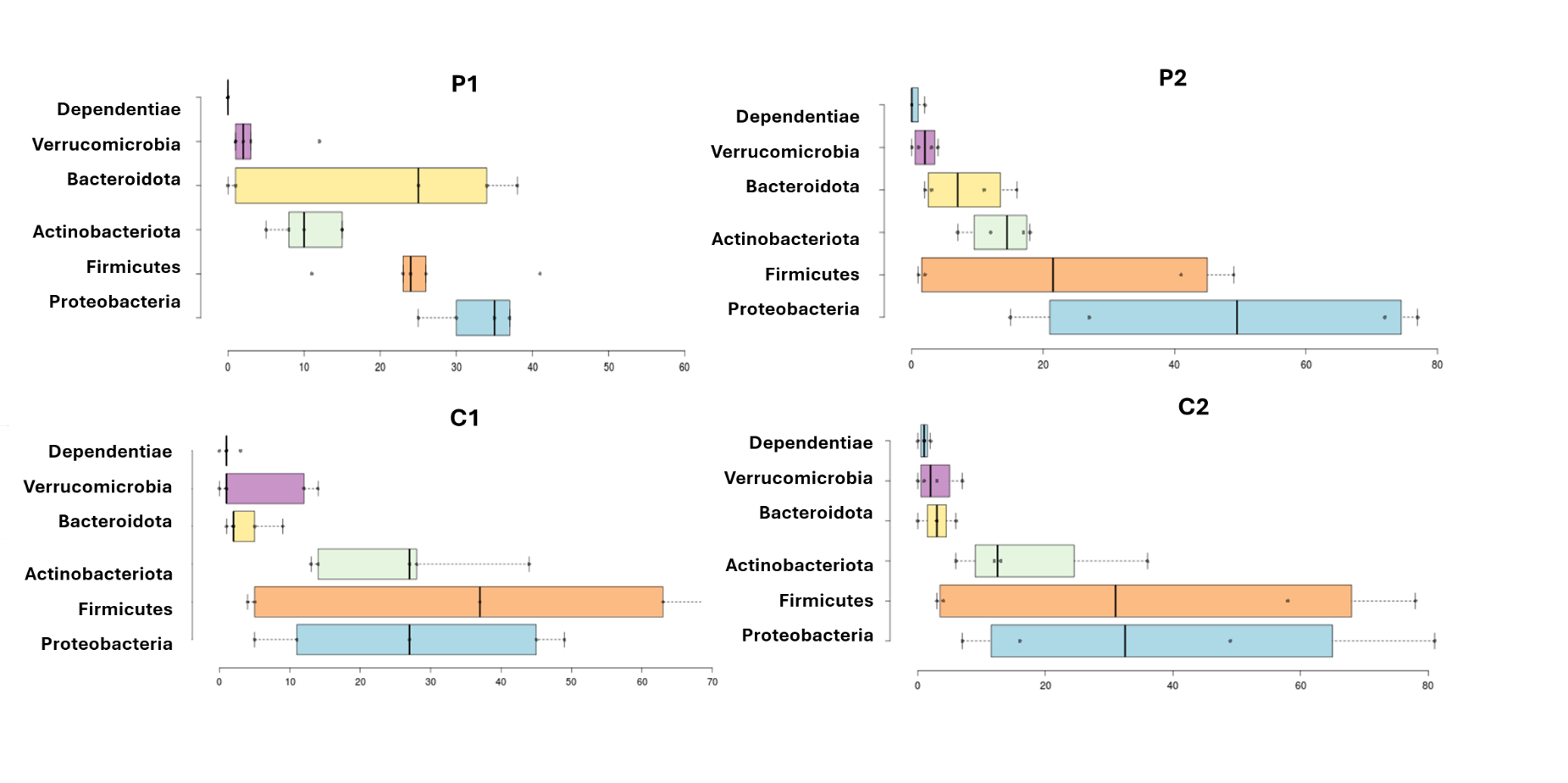
**Figure S1.** Taxonomic composition of microbial communities at the phylum level associated with *C. caretta* nests at Cala Pisana (P1, P2) and Spiaggia dei Conigli (C1, C2).
